# Evaluation of the holding-up uterus technique for placenta accreta spectrum cesarean hysterectomy in shocked patients with a high shock index: a case series study

**DOI:** 10.1186/s12893-024-02311-8

**Published:** 2024-01-13

**Authors:** Jin Takahashi, Makoto Orisaka, Daisuke Inoue, Hiroshi Kawamura, Nozomu Takahashi, Hideaki Tsuyoshi, Akiko Shinagawa, Tetsuji Kurokawa, Yoshio Yoshida

**Affiliations:** 1 Department of Obstetrics and Gynecology, University of Fukui, Fukui, Japan; 2 Department of Obstetrics and Gynecology, Fukui Prefectural Hospital, Fukui, Japan

**Keywords:** Placenta accreta spectrum, Cesarean hysterectomy, Shock index, Holding-up uterus technique, Preoperative intervention

## Abstract

**Background:**

Placenta accreta spectrum (PAS) cesarean hysterectomy is performed under conditions of shock and can result in serious complications. This study aimed to evaluate the usefulness of the “Holding-up uterus” surgical technique with a shock index (S.I.) > 1.5.

**Methods:**

Twelve patients who underwent PAS cesarean hysterectomy were included in the study.

**Results:**

Group I had S.I. > 1.5, and group II had S.I. ≤ 1.5. Group I had more complications, but none were above Grade 3 or fatal. Preoperative scheduled uterine artery embolization did not result in serious complications, but three patients who had emergency common iliac artery balloon occlusion (CIABO) and a primary total hysterectomy with S.I. > 1.5 had postoperative Grade 2 thrombosis. Two patients underwent manual ablation of the placenta under CIABO to preserve the uterus, both with S.I. > 1.5.

**Conclusions:**

The study found that the “Holding-up uterus” technique was safe, even in critical situations with S.I. > 1.5. CIABO had no intervention effect. The study also identified assisted reproductive technology pregnancies with a uterine cavity length of less than 5 cm before conception as a critical factor.

**Supplementary Information:**

The online version contains supplementary material available at 10.1186/s12893-024-02311-8.

## What does this study add to the clinical work

•The holding-up uterus technique following periuterine cavity expanded enables safe placenta accreta spectrum (PAS) cesarean hysterectomy.
•PAS hysterectomy with holding-up uterus is effective even in situation of critical bleeding or shock.

**Supplementary Information:**

The online version contains supplementary material available at 10.1186/s12893-024-02311-8.

## Introduction

Critical obstetric hemorrhage is a significant cause of maternal mortality, currently accounting for 27% of maternal deaths worldwide and is the leading cause of maternal mortality in Japan, accounting for 22% of maternal deaths [1, 2]. Because disseminated intravascular coagulation (DIC) can easily complicate even moderate amounts of blood loss, especially in cases of obstetric hemorrhage with underlying disease [3], a decision must be made to perform a pregnancy-related hysterectomy at the appropriate time. In particular, the proportion of pregnant women with an underlying condition of placenta accreta spectrum (PAS) has continued to increase with the recent increase in cesarean section deliveries, and the associated number of PAS cesarean hysterectomies has also increased. These findings are evident in data from two recently published large multinational cohort studies [4], in which PAS cesarean hysterectomies performed as the final step in a management protocol for massive hemorrhage associated with PAS disorders are associated with considerable maternal morbidity and mortality reportedly high. We need to be aware of this condition and the difficulties in its diagnosis and management and be prepared for the surgical procedure and its management.

Despite the increasing trend, the occasions when PAS cesarean hysterectomy must be performed are extremely rare. Therefore, many surgical procedures and general management algorithms have been proposed by quality centers with a multidisciplinary approach, but unfortunately, not all of them are based on pathologically confirmed cases of PAS [5]. Despite the high morbidity and mortality associated with hysterectomy, many studies unanimously suggest that it should be performed under multidisciplinary management. Recently, a multidisciplinary management algorithm has gained attention, proposing the involvement of gynecologic oncologists in the surgical management of PAS cesarean hysterectomies. The rationale behind this proposal is that the changes occurring in the female reproductive system during pregnancy add complexity to PAS cesarean hysterectomies [6].

The purpose of this study, which is a single-center, retrospective study, is to evaluate the diagnostic, surgical, and management of cases in which obstetricians and gynecologic oncologists performed multidisciplinary management and PAS cesarean hysterectomy, to determine the impact on maternal morbidity, and to help establish this troublesome treatment modality in the future.

## Materials and methods

### Cases and surgical procedures

A case series study of single, non-normal pregnancies pathologically confirmed as PAS in hysterectomized uteri between 2013 and 2022 at the University of Fukui. The primary endpoint was to assess intraoperative and postoperative complications associated with hysterectomy. The definition of complications was determined based on Clavien-Dindo classification [7]. Secondary endpoints were to examine preoperative diagnostic ability and risk factors for PAS. In addition, we examined and evaluate the utility of management interventions implemented in the sequence of events leading to obstetric crisis hemorrhage and PAS cesarean hysterectomy. We examined whether these endpoints differed between patients who underwent hysterectomy with a Shock Index (S.I.) > 1.5 at hysterectomy (Group I; S.I.>1.5) and those who underwent total hysterectomy before reaching this state (Group II; S.I. ≤ 1.5) [8, 9].

Our surgical procedure for PAS cesarean hysterectomy, while comparable to non-obstetric hysterectomy in general steps, exhibits distinctive characteristics that are tailored to the complexity of PAS. The operation is meticulously planned and executed by our multidisciplinary team, which is led by experienced gynecologic oncologists. The following outlines the full operative steps.

#### Preoperative planning and team assembly

A comprehensive preoperative plan is formulated, with a multidisciplinary team at the helm to ensure all necessary expertise is available. This team includes, but is not limited to, gynecologic oncologists, anesthesiologists, neonatologists, and urologists.

#### Vascular access and monitoring

Central venous access is established to facilitate rapid fluid administration and central venous pressure monitoring. An arterial line is placed for continuous blood pressure monitoring and regular blood gas analysis. The patient’s hemodynamic status is closely monitored throughout the procedure. Adjustments to medication regimens are made in real-time, based on ongoing assessments of blood loss, uterine tone, and the patient’s overall condition.

#### Anesthetic management

Anesthesia is initiated with either spinal or epidural anesthesia. In cases where a total hysterectomy is anticipated, the patient is transitioned to general anesthesia to ensure patient immobility and optimal pain control.

#### Patient positioning

The patient is positioned in the lithotomy position to provide the surgical team with adequate access to the operative field.

#### Hemostatic measures preparation

- Intrauterine balloon catheters (such as the Atom Uterine Compression Balloon) are prepared for potential rapid deployment to control uterine bleeding.
- Uterine compression sutures with blunt needles are on standby for immediate use if necessary.
- Iliac artery balloon occlusion is prepared in collaboration with the radiology department. Toe SpO2 monitors are also attached to monitor peripheral perfusion. When employed, the balloon is expanded for 15 min with 5-min intervals, without heparinization to mitigate the risk of bleeding.

#### Blood products and transfusion management

Transfusion preparations include the availability of autologous blood (4–8 units) and allogeneic blood with 10 units of red blood cells and 10 units of fresh frozen plasma, ensuring readiness for potential massive blood loss.

#### Insertion of bilateral ureteral stents

In all planned surgeries, bilateral ureteral stents are inserted preoperatively. This step is crucial for the identification and preservation of the ureters during the surgery.

#### Uterine incision and fetal extraction

The uterus is typically incised transversely, unless otherwise indicated by the placental position. The method of fetal extraction is determined by fetal position and placental location, with care taken to avoid placental disruption.

#### Administration of ecbolics and other medications

Immediately after the delivery of the fetus, ecbolics are administered to control bleeding. Intravenous oxytocin is administered as the first-line agent. A 10 units of bolus dose is given initially, followed by a continuous infusion to sustain uterine contractions. The dose is titrated based on the response of the uterus and the clinical judgment of the attending anesthesiologist and obstetrician. The use of additional ecbolics, such as methylergonovine or carboprost, is considered if the response to oxytocin is inadequate or if there is a contraindication to its use. The selection of these agents is tailored to the individual’s clinical status, including blood pressure and any pre-existing medical conditions.

Simultaneously, the uterine incision wound is quickly sutured simply to hemostat and promote uterine contractions.

#### Use of energy devices

Energy devices such as Bipolar scissors (Ellman-Japan, Osaka, Japan) and HARMONIC FOCUS® (ETHICON, Bridgewater, NL, USA) are employed for cutting and coagulation, which minimizes blood loss and enhances precision in tissue dissection. The upper uterine ligament is fully ligated and severed. The ovaries are typically preserved.

#### Bladder and uterine cavity preparation

To facilitate a complete hysterectomy, the lateral cavity of the bladder is meticulously expanded, and the lateral cavity of the uterus is secured before dissection begins. This preparation is critical for safely accessing the surgical planes.

#### Cystocele dissection and release of the uterus

Following the separation of the bladder, the sacrouterine ligaments are transected, and the Douglas fossa peritoneum is incised to release the uterus posteriorly and laterally.

#### The “holding-up uterus” method

Our unique ‘holding-up uterus’ technique is then employed. The surgeon places one hand in the cysto-uterine fossa and the other in the Douglas fossa to grasp and lift the entire uterus. This method not only provides superior visualization but also creates necessary distance between the ureter and the cervix, facilitating the safe liberation of the ureter.

#### Identification and division of uterine artery

With the uterus lifted, the uterine artery can be clearly identified and safely divided. This step is critical to controlling the blood supply to the uterus and ensuring hemostasis.

#### Completion of hysterectomy

The cervix is amputated at the predetermined site, identified by inserting a finger into the posterior fornix and lifting the cervix. The uterus is then completely removed.

#### Hemostasis verification

A thorough examination of the surgical field is conducted to confirm complete hemostasis. If any bleeding points are identified, they are addressed immediately with additional sutures, electrocautery, or application of hemostatic agents as required. The bilateral uterine arteries and the site of the cervix, particularly, are inspected meticulously given their potential as primary sources of hemorrhage.

#### Application of adhesion prevention agents

Once hemostasis is confirmed, adhesion prevention agents are applied. These may include hyaluronic acid-based gels or oxidized regenerated cellulose, which are strategically placed over areas prone to adhesion formation, such as the raw surfaces created by dissection. This step is crucial for reducing the risk of postoperative adhesions, which can lead to chronic pain and ileus.

**Fig. 1:**
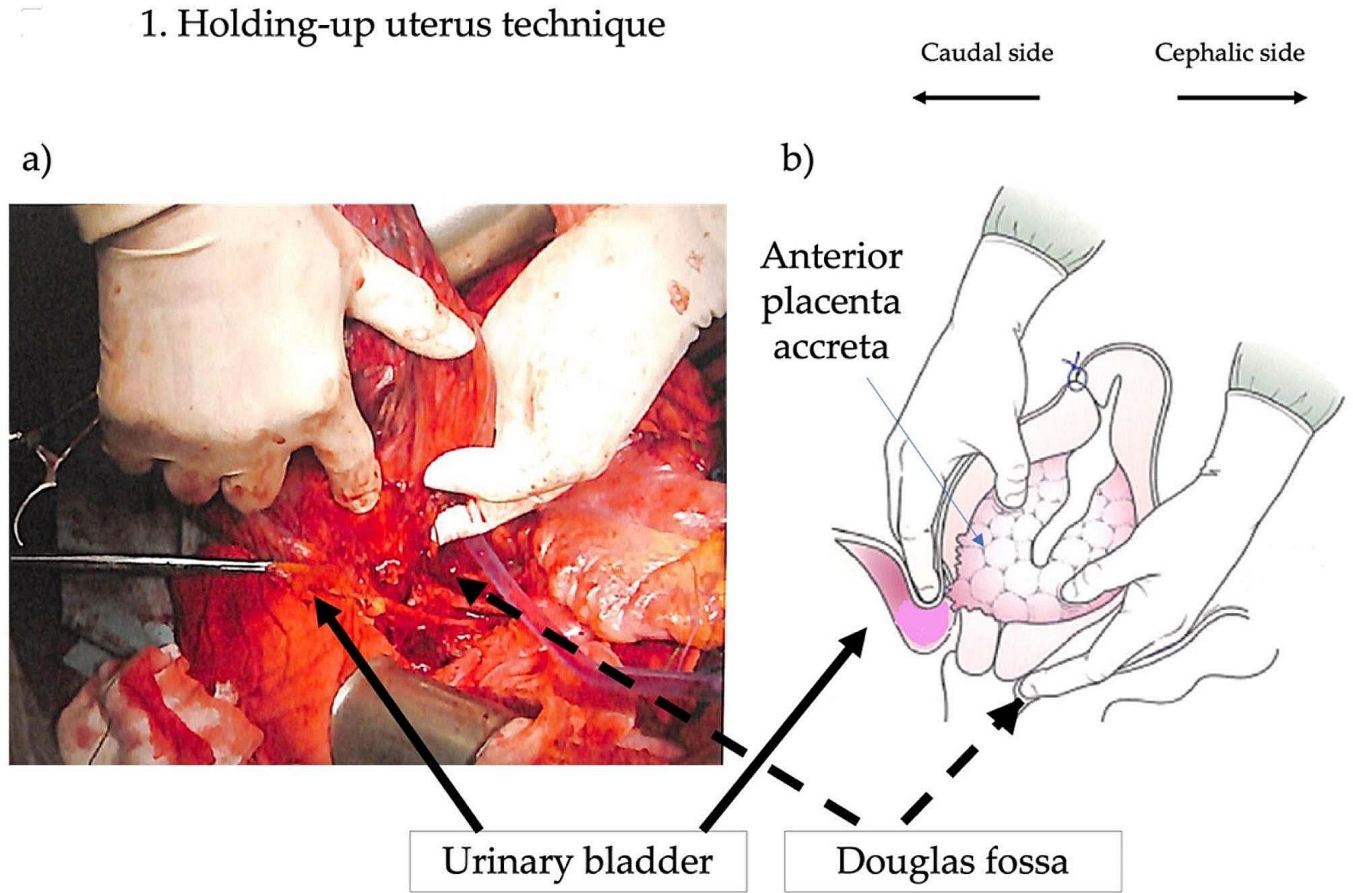
(**a**) Shows the “Holding-up uterus” technique in the PAS cesarean hysterectomy. (**b**) is reproduced from Teiou sekkai no kyoukasyo by Yoshida et al. published in KANEHARA & Co., LTD, pp. 163, 2017

Each step is conducted with utmost precision, keeping in mind the altered anatomy due to PAS. Our technique is notable for the emphasis on preoperative stenting of the ureters, the use of advanced energy devices, and the strategic ‘holding-up uterus’ maneuver, all of which are pivotal for the success of these complex surgical procedures. (Fig. 1 and Additional file 1)

### Statistical analysis

Continuous variables were described using mean ± standard deviation. To evaluate normally distributed data, the student t-test was utilized, and Mann-Whitney’s U test was used for between-group comparisons to evaluate data that were not normally distributed. PAS and diagnostic efficiency in ultrasonography and MRI examinations were performed with the binomial distribution test, and significance between the two was performed with Fisher’s exact definite test. The IBM Statistical Package for the Social Sciences (SPSS, version 22, SPSS Inc., Chicago, IL, USA) software was used in all analyses. P-values < 0.05 were judged as significant.

## Results

Twelve patients who were managed conservatively for obstetric crisis hemorrhage at the University of Fukui Hospital from 2013 to 2023, but who ultimately underwent PAS caesarean hysterectomy. Pathology included four cases of simple adherent placenta (FIGO Grade 1), seven cases of invasive placenta (FIGO Grade 2) and one case of placental penetration (FIGO Grade 3) (Sup. Tables 1, 2) [10].

The group that underwent total hysterectomy with S.I. > 1.5 (Group I) had 6 cases with a total blood loss of 5490 mL (± 1821 mL), and the group that underwent total hysterectomy by S.I. ≤ 1.5 (Group II) had a total blood loss of 1959 mL (± 909 mL). With regard to intraoperative and postoperative complications, there were no serious complications above Grade 3 or deaths in Group I, although significantly more complications occurred in Group I. The only intraoperative complication was a partial bladder injury: one in Group I and the other in Group II. Both were completely healed by surgical repair sutures at the same time (Table 1).

**Table 1:** Intraoperative and postoperative complications associated with hysterectomy

	Group 1 S.I. > 1.5 (*n* = 6)	Group 2 S.I. ≤ 1.5 (*n* = 6)		P-value
Total blood loss, including during hysterectomy (mL)*	5490 ± 1821		1959 ± 909	
Intraoperative Grade 2 or higher adverse events**	1 (16)		1 (16)	0.104
Postoperative Grade 2 or higher adverse events**	4 (16)		2 (33)	0.018
Hospitalization for more than 7 days after hysterectomy**	6 (33)		1 (16)	0.001
FIGO stage**	Grade1; 2 Grade2; 3 Grade3; 1		Grade1; 2 Grade2; 4	0.664

S.I.: Shock index * Data presented as Mean ± SD. **Data presented as n (%). Independent sample *t* test

None of the three cases in which PAS was suspected preoperatively and combined with uterine artery embolization (UAE) had any serious complications. In addition, a total hysterectomy with UAE performed immediately after cesarean section and additional prophylactic intravascular balloon catheter placed within 7 days after surgery was safe. Three out of three patients who underwent emergency common iliac artery balloon occlusion (CIABO) and then primary total hysterectomy with S.I. > 1.5 developed postoperative Grade 2 thrombosis. Three patients in group I had preoperative suspicion of placenta accreta and underwent scheduled surgery, and three patients had no suspicion of placenta accreta and underwent emergency total hysterectomy. Three patients in group II also had preoperative suspicion of placenta accreta, while three had no suspicion of placenta accreta. Preoperative intervention for obstetric crisis hemorrhage due to PAS was more common in group I, and intervascular radiology was comparable in both groups (Table 2).

**Table 2:** Interventions for preoperative treatment of obstetric crisis hemorrhage by placenta accreta spectrum

	Group 1 S.I. > 1.5 (*n* = 6)	Group 2 S.I. ≤ 1.5 (*n* = 6)	P-value
Medications (uterotonic agents)			
oxytocin*	5 (83)	3 (50)	0.339
**Surgical Intervention**			
Interventional radiology*	4 (66)	3 (50)	0.339
Intrauterine balloon tamponade*	3 (50)	0	0.082
Uterine compression suture*	4 (66)	0	0.066
Vaginal/uterine packing *	2 (33)	2 (33)	0.438
Placental bed suture*	3 (50)	0	0.082

* Data presented as n (%). Independent sample *t* test

At our institution, initial screening was performed with ultrasound, and MRI was the second imaging modality of choice when PAS was suspected. In the present study, there were 6 suspected cases on ultrasound and 4 suspected cases on MRI. The present study did not predict the diagnosis of PAS or cases with S.I. > 1.5 on risk factor assessment or preoperative imaging evaluation. The diagnostic efficiency of ultrasound and MRI examinations performed at our institution with PAS was 0.5 (50%), with a binomial distribution binomial distribution test, the point estimate for preoperative diagnosis of PAS by ultrasound was 0.5 (50%), with a 95% confidence interval of 0.218 (21.8%) to 0.782 (78.2 (%). For MRI, the rate was 0.444 (44.4%), with 95% confidence intervals ranging from 0.121 (12.1%) to 0.767 (76.7%). There was no difference in diagnostic efficiency between the two, with a P value = 0.3306 in Fisher’s exact definite test.

The history of the patients in the present study was characterized by the fact that one patient had undergone three cesarean sections and two patients had undergone one cesarean section. Six patients had total placenta previa. Other surgical procedures that caused surgical damage to the uterine wall, such as surgical hysteroscopy, and suction curettage were observed in three cases. Notable history includes one patient with a history of UAE, two patients with a uterine cavity length of less than 5 cm at non-pregnancy, and two patients with systemic lupus erythematosus [11]. In vitro fertilization (using cryopreserved embryos) was performed in 7 cases. The only significant difference was that 2 assisted reproductive technology (ART) pregnancies with a pre-pregnancy uterine cavity length of less than 5 cm were observed in Group I (Table 3). There was no significant difference between the two groups in pre-operative management, but there was more pre-operative management in Group I (Table 2).

**Table 3:** Preoperative risk factors for placenta accreta spectrum

	Group 1 S.I. > 1.5 (*n* = 6)	Group 2 S.I. ≤ 1.5 (*n* = 6)	P-value
Age*	35.7 ± 7.14	36.0 ± 4.6	
History of cesarean section**	1 (16)	2 (33)	0.809
Abortion**	2 (33)	1 (16)	0.191
History of intrauterine curettage**	2 (33)	1 (16)	0.191
ART**	4 (67)	4 (67)	0.438
History of placental delivery difficulty**	1 (16)	1 (16)	0.104
History of SLE**	1 (16)	1 (16)	0.104
Pre-pregnancy uterine cavity length is less than 5 cm**	2 (33)	0 (0)	0.039

ART: assisted reproductive technology; SLE: systemic lupus erythematosus; ARDS: Acute respiratory distress syndrome; NA: Not available

* Data presented as Mean ± SD. **Data presented as n (%). Independent sample *t* test

## Discussion

Our “Holding-up uterus” method during PAS cesarean hysterectomy, even in critical situations with S.I. >1.5, facilitates ureteral emancipation, identification of the uterine artery, and facilitates the Pelosi method [12] in which the bladder is finally detached after cutting the vaginal wall, avoiding adhesions on the anterior bladder from the previous cesarean-section. In cases of severe adhesions, the posterior vaginal canal may be opened first, facilitating the treatment of the basal ligament and dissection of the bladder and anterior vaginal wall, which may be an extremely useful method for complete hysterectomy.

PAS cesarean hysterectomy has become the gold standard as the final step in the management protocol for massive hemorrhage associated with PAS disorders. However, this primary radical surgical treatment is associated with a high incidence of maternal surgical-related adverse events, particularly massive hemorrhage, and damage to surrounding organs (40–50%) and maternal death (approximately 7%) [13, 14]. Pregnancy-related hysterectomy for PAS is considerably more technically challenging than hysterectomy for uterine atony because of the higher risk of adjacent organ injury [15]. Urinary tract injuries have been reported in 29% of surgeries, with lacerations of the bladder reported in 76%, ureteral injuries in 17%, and urogenital fistulas in 5% [16].

The procedure for pregnancy-related hysterectomy is identical to that for non-pregnancy hysterectomy [17]. However, during the operation, one must be aware of the changes that occur in the female reproductive organs during pregnancy [17]. As the uterus enlarges, it becomes more difficult to manipulate it and to visualize the entire pelvis. In addition, the ureters may become tortuous and dilated, resulting in significant hydroureteria. Tissue fragility and edema increase. Most importantly, uterine blood flow increases 10- to 30-fold in late pregnancy, and pregnant women with underlying diseases such as PAS are more prone to complications of DIC, even with moderate blood loss [3]. The reason is that it has been reported to shorten operative time and decrease blood loss. We use energy devices during hysterectomy. The reason is that it has been reported to reduce operative time and blood loss [18]. And supra-hysterectomy is often performed because of the short operating time required under conditions of critical bleeding, the increased risk of ureteral injury during emergency surgery, and, in the case of placenta previa, the fully dilated cervix, which makes identification of the transition from the cervix to the vagina difficult. However, FIGO recommends performing a supra-hysterectomy because of the potential risk of malignancy in the residual cervix and the consequent need for periodic cervical cytology, and because the residual cervix is a cause of postoperative bleeding (placenta previa PAS) [19]. It has also recently been shown that identification and clamping of the bilateral uterine arteries and removal of the uterus as low as possible at the inferior margin of the placenta, avoiding the ureter, reduces maternal bleeding morbidity the most [20].

For this reason, we try to perform total hysterectomies using the “Holding-up uterus” technique even in emergency situations. In this context, our “Holding-up uterus” technique during PAS cesarean hysterectomy facilitates ureteral emancipation, identification of the uterine artery, and dissection of the adherent bladder. Currently, even in critical situations of Group I (S.I. > 1.5), the operation can be performed in a short time. One case of bladder injury was observed in each Group I and II, but it could be easily repaired.

Another proposed radical surgery is delayed hysterectomy. In this procedure, the uterus is closed after delivery, leaving the placenta in uterus, the mother’s abdomen is closed, and then a total hysterectomy is performed 3–12 weeks later. The rationale for this procedure is that uterine perfusion is reduced after delivery, even if the placenta is left in uterus, and the subsequent surgery is less risky for the woman due to uterine retraction and decreased vascularity [21]. However, abdominal closure for the purpose of delayed total hysterectomy may be followed by massive bleeding due to partial abruption of the placenta.

Prophylactic intravascular balloon catheters have been proposed to reduce intraoperative bleeding during hysterectomy. It improves maternal morbidity and allows the surgeon to operate in a “cleaner” and more visible operative field. However, the incidence of potential complications is high, including the risk of vessel rupture, thromboembolism development, and impaired blood supply to the lower extremity [22]. In addition, PAS is associated with extensive abnormal neovascularization, and occlusion of some pelvic vessels may increase blood loss from collateral blood vessels [6, 22, 23]. Furthermore, two RCTs comparing balloon catheter placement in the iliac artery versus no intervention at all found no difference in the number of packed red blood cells transfused to patients, and a recent RTC comparing bilateral internal iliac artery ligation versus control found no difference regarding intraoperative blood loss [6, 22, 23]. In our study, in the critical situation of group I (S.I. >1.5), total hysterectomy with intravascular balloon insertion was more likely to cause postoperative venous thrombosis.

The most important risk factor for the development of PAS has been shown to be the number of previous cesarean Sect. [6]. In the present study, one patient had had three cesarean sections and two had had one cesarean section. In vitro fertilization (using cryopreserved embryos), a risk factor that has received much attention recently, was used in seven cases. In terms of preoperative assessment factors, 67% of pregnancies in both groups were ART pregnancies. The only significant difference was that two ART pregnancies with hypoplastic uteri were observed in Group I. Although the definition of hypoplastic uterus is not known, both pregnancies were ART pregnancies with small pre-eclamptic uteri and a cervix to uterus size ratio of 1:1. The uterine cavity length was about 5 cm.

There was no difference in diagnostic efficiency between PAS and diagnostic efficiency between ultrasound and MRI examinations performed at our hospital. This result did not differ significantly from the prenatal detection rate of PAS by ultrasound in two large population-based studies in the U.K. and U.S [24, 25]. Nearly half of PAS is diagnosed only at birth, and even among pregnant women who underwent prenatal MRI testing, over a quarter were detected at birth [17]. The inclusion of imaging and clinical factors has been reported to improve the prenatal diagnosis of PAS [17] and should be actively investigated in the future.

“Holding-up uterus” method has shown several significant strengths in the management of PAS cesarean hysterectomy. Firstly, it has proven to be beneficial in providing improved operative visualization. By holding up the uterus, the technique allows for a clearer view of the ureters and uterine arteries, which is crucial in the context of PAS where normal anatomical landmarks may be distorted. This is of particular importance as it facilitates the Pelosi method and other critical steps such as ureteral emancipation. Moreover, the method has been associated with reduced operative time and blood loss, which are critical outcomes in PAS cesarean hysterectomy. This reduction is not only beneficial for the patient’s immediate surgical outcome but also has long-term implications on their recovery process. The facilitation of complex surgical steps such as the dissection of the adherent bladder and identification of the uterine artery is another advantage that cannot be overstated. This simplification is vital, especially in the backdrop of the high incidence of maternal surgical-related adverse events. The ability of the technique to be effectively utilized even in emergency situations where the shock index is high is a testament to its robustness. This is underscored by our findings that even in group I (S.I. > 1.5), the operation can be executed in a short time frame, indicating that the method is adaptable to critical situations. Additionally, the practice of performing total hysterectomies aligns with recommendations to reduce potential risks associated with residual cervical tissue.

However, the limitations of our technique are as important to consider as its strengths. Organ injury remains a substantial risk in PAS cesarean hysterectomy, and while our method aids in minimizing this risk, it does not eliminate it. The surgeon’s experience and the technique’s learning curve are additional factors that could affect the outcomes of the surgery. Our findings also suggest that the generalizability of the “Holding-up uterus” method might be limited by variations in surgical practices across different institutions. Furthermore, the comparative data on the “Holding-up uterus” method versus other techniques is not extensive. This lack of robust comparative data could be seen as a limitation as it does not allow for a conclusive argument for the superiority of our method. While we have observed benefits in our own practice, additional comparative studies are required to validate these findings further. Lastly, despite the method being designed to minimize complications, the potential for unforeseen surgical difficulties and postoperative complications remains. This highlights the necessity of vigilance and preparedness for managing such events should they occur.

While our “Holding-up uterus” method demonstrates considerable promise, particularly in facilitating ureteral emancipation, identification of the uterine artery, and dissection of the adherent bladder, there is a need for a careful evaluation of the risks and benefits. Future studies should aim to provide a more comprehensive comparison with other techniques, evaluate the learning curve associated with the method, and explore strategies to mitigate the inherent risks of PAS cesarean hysterectomy.

## Conclusions

The primary surgical treatment for PAS is pregnancy-related hysterectomy, which is technically challenging due to the increased risk of adjacent organ injury. The “Holding-up uterus” technique during PAS cesarean hysterectomy facilitates ureteral emancipation, identification of the uterine artery, and dissection of the adherent bladder, making the total hysterectomy easier to perform even in critical situations. Prophylactic intravascular balloon catheters have been proposed to reduce intraoperative bleeding during hysterectomy, but their use may lead to potential complications. The most important risk factor for the development of PAS is the number of previous cesarean sections.

Future research should focus on collecting high-quality data from well-designed prospective studies on a multidisciplinary team approach to diagnosis (prenatal imaging) and management strategies.

### Electronic supplementary material

Below is the link to the electronic supplementary material.

**Supplementary Material 1: Supplementary Table1.** Case series

**Supplementary Material 2**

**Supplementary Material 3: Supplementary Table2.** Case series

## Data Availability

The data presented in this study are available in Tables 1, 2 and 3 and supplemental Tables 1, 2.
