# Supplementary material for: Evaluation of the holding-up uterus technique for placenta accreta spectrum cesarean hysterectomy in shocked patients with a high shock index: a case series study
Source: BMC Surg. 2024 Jan 13;24:23. doi: 10.1186/s12893-024-02311-8 (PMC10787967; doi:10.1186/s12893-024-02311-8)
Supplement: Supplementary file 1 — Supplementary Material 1: Supplementary Table1. Case series [file 12893_2024_2311_MOESM1_ESM.docx]

Supplementary Table1. Case series

| **No.** | **Age**  **Gravida/Para** | **Gestational weeks at C/S** | **Pregnancy method** | **Medical history** | **Suspicious of PAS** | | **Placental position** | **Pathological**  **diagnosis**  **(FIGO)** |
| --- | --- | --- | --- | --- | --- | --- | --- | --- |
|  |  |  |  |  | **US** | **MRI** |  |  |
| 1 | 28, G4P3 | 36-4 | Natural | Previous C/S (3 times) | + | + | placenta previa | 2 |
| 2 | 40, G2P1 | 36-4 | ART | Previous C/S (1 time) | + | + | placenta previa | 2 |
| 3 | 36, G2P1 | 34-1 | Natural | Previous C/S (1 time) | + | + | placenta previa | 1 |
| 4 | 35, G2P1 | 33-4 | Natural | History of conservative treatment of retained placental in uterine artery embolization  (History of placental delivery difficulties) | + | + | placenta previa | 2 |
| 5 | 42, G2P0 | 37-3 | ART | 1 intrauterine curettage | + | - | placenta previa | 2 |
| 6 | 33, G2P1 | 33-2 | Natural | Massive bleeding (1700 mL) at last vaginal delivery  (History of placental delivery difficulty) | + | - | placenta previa | 2 |
| 7 | 45, G1P0 | 35-4 | ART  (Frozen embryo) | Endometrial polypectomy (trance cervical resection)  Removal of uterine contents by manual vacuum aspiration  Transverse colon resection (external endometriosis) | - | - | placenta previa at the margins | 2 |
| 8 | 40, G1P0 | 35-2 | ART  (Frozen embryo) | SLE | - | - | Back wall to right side wall adhesion | 2 |
| 9 | 39, G3P1 | 39-2 | Natural | 1 septic ARDS intrauterine curettage with intra uterine fetal death  Bilobed uterus | - | - | Back wall adhesion | 1 |
| 10 | 34, G2P1 | 39-1 | ART  (Frozen embryos) |  | - | NA | Bottom to right posterior wall adhesion | 1 |
| 11 | 33, G1P0 | 41-2 | ART  (Frozen embryo) | Uterine hypoplasia (uterine cavity length 5.5 cm)  Hysteroscopy | - | NA | back wall adhesion | 1 |
| 12 | 25, G1P0 | 37-0 | ART  (Frozen embryo) | SLE  Uterine hypoplasia (uterine cavity length 5 cm) | - | NA | Back wall to front wall adhesion | 3a |

C/S: Cesarean section; PAS: Placenta accreta spectrum; US: Ultrasonography; MRI: Magnetic resonance imaging; ART: assisted reproductive technology; SLE: systemic lupus erythematosus; ARDS: Acute respiratory distress syndrome; NA: Not available
