# Supplementary material for: Evaluation of the holding-up uterus technique for placenta accreta spectrum cesarean hysterectomy in shocked patients with a high shock index: a case series study
Source: BMC Surg. 2024 Jan 13;24:23. doi: 10.1186/s12893-024-02311-8 (PMC10787967; doi:10.1186/s12893-024-02311-8)
Supplement: Supplementary file 3 — Supplementary Material 3: Supplementary Table2. Case series [file 12893_2024_2311_MOESM3_ESM.docx]

Supplementary Table2. Case series

| **No.** | **Placental abruption** | **Pre-hysterectomy management** | **Status of total hysterectomy** | **Amount of blood loss** | **Intraoperative drug use** | | **Intraoperative**  **adverse event** | **Postoperative**  **adverse event** | **S.I.**  **Group** |
| --- | --- | --- | --- | --- | --- | --- | --- | --- | --- |
|  |  |  |  |  | **Ecbolic** | **Others** |  |  |  |
| 1 | Normally | UAE performed after C/S (Day 0)  Second UAE enforcement (Day 7) | Delayed hysterectomy (Day 8) | 1,030 mL at C/S (Day 0)  330 mL at hysterectomy (Day 8) | Oxytocin  20 Units | Phenylephrine-hydrochloride | - | - | II |
| 2 | Normally | Vaginal/uterine packing  Post C/S UAE (Day 0) | Delayed hysterectomy (Day 5) | 2,670mL at C/S (Day 0)  190 mL at hysterectomy (Day5) | Oxytocin  30 Units | Phenylephrine-hydrochloride  Antithrombin-gamma | - | - | II |
| 3 | Normally | CIABO performed after C/S | One-stage hysterectomy under CIABO | 2500 mL at C/S  800 mL at hysterectomy | Oxytocin 40 Units | Phenylephrine-hydrochloride | Bladder injury | ovarian vein thrombus, Grade 2  Appearance of numbness in right thumb, Grade 2 | I |
| 4 | Normally | UAE performed after C/S (Day 0) | Delayed hysterectomy under CIABO (Day 1) | 388 mL at C/S (Day0)  645 mL at hysterectomy (Day 1) | Oxytocin  10 Units | Phenylephrine-hydrochloride | - |  | II |
| 5 | Manual removal during C/S under CIABO | Placental bed suture  Uterine compression suture  Intrauterine balloon tamponade | One-stage hysterectomy  Strong bleeding persistence even with CIABO | 4,576mL | Oxytocin  65 Units | Phenylephrine-hydrochloride | - | Intestinal obstruction, Grade 2 | I |
| 6 | Manual removal during C/S under CIABO | Placental bed suture  Uterine compression suture  Intrauterine balloon tamponade | One-stage hysterectomy  Strong bleeding persistence even with CIABO | 6,534mL | Oxytocin  45 Units | Phenylephrine-hydrochloride  Noradrenaline  Human anti-thrombin III | - | Intra-abdominal abscess, Grade 2  Pulmonary embolus and ovarian vein thrombus, Grade 2 | I |
| 7 | Manual removal | Placental bed suture  Uterine compression suture | One-stage supra-hysterectomy with SI > 1.5 | 8,239mL | Oxytocin  75 Units | Phenylephrine-hydrochloride | - | - | I |
| 8 | Normally |  | One-stage hysterectomy | 1,110mL | Oxytocin  10 Units  Methylergometrine maleate 0.2mg | Phenylephrine-hydrochloride  Human anti-thrombin III | - | - | II |
| 9 | Normally | Vaginal/uterine packing  Cervical laceration suture and laceration reattachment (Day 5) | Delayed hysterectomy (Day 8) | 1,982mL at delivery  1,010mL at Day 5  200 mL at hysterectomy (Day 8) | Oxytocin  40 Units | Phenylephrine-hydrochloride | Bladder injury | Transection dissection, Grade 2  Bowel obstruction, Grade 2 | II |
| 10 | Manual removal  but retained placenta |  | Delayed hysterectomy (Day 12)  due to infection of retained placenta | 1,194 ml at delivery  220mL at hysterectomy | Oxytocin  80 Units | Phenylephrine-hydrochloride | - | - | II |
| 11 | Manual removal | Placental bed suture  Intrauterine balloon tamponade/vaginal/uterine packing | One-stage hysterectomy  (Urgent after UAE failure) | 6,168mL at delivery  296 mL at hysterectomy | Oxytocin 50 Units Methylergometrine maleate 0.2mg | Phenylephrine-hydrochloride  Noradrenaline | - | - | I |
| 12 | Normally | Vaginal/uterine packing | One-stage hysterectomy | 4,127mL | Oxytocin  40 Units | Phenylephrine-hydrochloride | - | - | I |

C/S: Cesarean section; PAS: Placenta accreta spectrum; US: Ultrasonography; MRI: Magnetic resonance imaging; ART: assisted reproductive technology; SLE: systemic lupus erythematosus; ARDS: Acute respiratory distress syndrome; NA: Not available
